# Adolescent school retention post COVID-19 school closures in Kenya: A mixed-methods study

**DOI:** 10.1371/journal.pone.0315497

**Published:** 2024-12-12

**Authors:** Ruth Nanjekho Wafubwa, Erica Soler-Hampejsek, Eva Muluve, Daniel Osuka, Karen Austrian

**Affiliations:** 1 Population Council—Kenya, Nairobi, Kenya; 2 Independent Consultant, Barcelona, Spain; 3 African Population Health Research Centre, Nairobi, Kenya; 4 GIRL Center, Population Council, Nairobi, Kenya; IFPRI: International Food Policy Research Institute, UNITED STATES OF AMERICA

## Abstract

This mixed methods study investigated factors associated with school retention among marginalized adolescents in four different settings in Kenya, following COVID-19 school closures. Logistic regressions were used to examine factors associated with school retention in 2022 among 1798 adolescent students aged 10–19 in 2020. Qualitative data from 89 in-depth interviews (64 adolescents aged 11–19 and 25 parents), and 21 key informants were thematically analysed. Among female adolescents, age (aOR = 0.76, 95% CI: 0.67, 0.87) and internet access (aOR = 0.55, 95% CI: 0.35, 0.87) were negatively associated with school retention. Engaging in income-generating activities was similarly linked to reduced school retention (aOR = 0.27, 95% CI: 0.16, 0.46). For male adolescents, household loss of income (aOR = 0.40, 95% CI: 0.21, 0.76) and engaging in income-generating activities (aOR = 0.07, 95% CI: 0.02, 0.19) were associated with lower school retention. The qualitative findings highlighted the gendered nature of barriers to school retention. Specifically, pregnancy, child marriage, and related childcare responsibilities emerged as important constraints for girls, whereas engaging in income-generating activities and drug and alcohol use were more dominant factors for boys. Across both genders, financial constraints were a key barrier to school retention. This study underscores the multifaceted nature of factors influencing school retention among marginalized adolescents in times of crisis such as the COVID-19 pandemic. The findings provide useful information for designing targeted policies and programmes for adolescent school retention in times of crisis.

## Background

The COVID-19 pandemic had far-reaching implications for education systems worldwide. Governments implemented a range of mitigation measures to contain the spread of the virus, including the temporary closure of schools [1, 2]. While these measures aimed to safeguard public health, they disrupted the education of millions of students globally, with those from most marginalized backgrounds (low-income families, rural communities, and those living in urban informal settlements, who already face significant barriers to accessing education) feeling the strongest implications. Close to 200 countries reported country-wide school closures affecting about 91% of school-going learners [3]. This education disruption had both short- and longer-term effects.

In Kenya, the study country, schools were closed for nearly 10 months at the height of the COVID-19 pandemic, causing significant disruption to the academic calendar, programmes, and term times. This in turn impacted the learning process and limited students’ access to quality education while exacerbating existing inequalities [4]. For example, marginalized adolescents such as those from low-income households, rural and remote areas, and vulnerable communities, faced greater challenges in continuing their education during the school closures [5]. This was exacerbated by limited access to technology, lack of internet connectivity, and poor access to mainstream media which sometimes offered educational programs [5–7].

School drop-out was among the more immediate effects of COVID-19 school closures [8]. Studies have documented several factors that were associated with school dropout during the first year of the COVID-19 pandemic. These included extended school closures and increased household economic stress [1, 9]. Parental loss of income during the pandemic also meant that marginalized adolescents had to take up income-generating activities to supplement household income, often impacting their ability to prioritize education and disrupting schooling [1, 10]. Reducing dropout rates and promoting school retention are critical goals in education policy to ensure that all students can complete their education and pursue their future goals and aspirations [11]. School retention is also a crucial indicator of educational success and is often used to assess the effectiveness of education systems [12, 13].

Marginalized girls in particular faced unique challenges due to the pandemic. For instance, the impact of COVID-19 on early pregnancy and child marriage was projected to reach 10 million globally [14]. In Kenya, COVID-19 school closures were indicated to affect adolescent girls more than boys. A study by the Presidential Policy and Strategy Unit (Kenya) and Population Council in 2021 [15], for example, showed that in Kenya, 16% of girls and 8% of boys aged 10–19 years who were in school prior to COVID-19 school closures, had not re-enrolled back. Another study [16] among secondary school girls in Western Kenya, showed that girls who were out of school because of the COVID-19 lockdowns had twice higher risk of getting pregnant and dropping out of school; compared to those who had graduated before the pandemic.

The short-term effects of COVID-19 school closures have been extensively studied [17–19]. However, the longer-term implications, particularly for marginalized adolescents, require further investigation. This study aimed to contribute to addressing this gap in knowledge by exploring factors related to school retention among marginalized adolescents residing in urban informal settlements and rural settings, two years after the onset of the pandemic. For this study, school retention was defined as students aged between 10–19 who were in school before the COVID-19 pandemic in March 2020, and were still in school by March 2022. Understanding the factors associated with school retention during and after a crisis such as a pandemic is important in designing targeted education interventions and policies; especially to support vulnerable groups. Additionally, this study integrates a gender-sensitive analysis to highlight disparities in retention between boys and girls, addressing gaps in previous research that often overlook the gendered dimensions of educational outcomes. The findings of this study can contribute to the development of effective strategies to mitigate the adverse effects of crises such as pandemics and ensure a more inclusive and equitable education system.

## Methods

### Study sites

This mixed methods study combined both quantitative and qualitative approaches and primarily drew on existing cohorts of vulnerable adolescents in four counties (Nairobi, Wajir, Kilifi, and Kisumu) in Kenya. These cohorts were from ongoing (pre-pandemic) impact evaluation studies of girls’ empowerment programs. The adolescent boys were sampled from the households with the sampled girls who were either their siblings, step-siblings, or cousins.

The four counties represent diverse geographic settings. Nairobi is an urban cosmopolitan city and the capital city of Kenya. In this county, the focus was on five urban informal settlements known as Kibera, Mathare, Dandora, Huruma, and Kariobangi. These urban informal settlements (colloquially referred to as ‘slums’) are characterized by very high population density and overcrowding, limited access to clean water, shared sanitation facilities, high poverty and unemployment rates, and pervasive food insecurity [20, 21]. These ‘slums’ tend to be a melting pot of various cultures and ethnicities from various parts of Kenya, with highly transitory residents.

Wajir County is a rural and very remote part of Kenya, categorized as arid and semi-arid lands (ASAL). The residents of Wajir are predominantly pastoralists of Somali ethnicity, with Islam being the primary religion. Except for Wajir town, dwellings in this county tend to be located several kilometres apart and built with semi-permanent materials such as thatched grass. It is also one of the most underdeveloped and socially conservative counties in Kenya; with low school enrolment rates (especially for girls), high rates of child marriage, and the accepted practice of female genital cutting (FGM) [22].

The third county, Kilifi, with a largely rural interior is located along the Kenyan coast. Residents of Kilifi are predominantly from the Giriama-speaking community and largely practice small-scale farming. Kilifi, as with many other coastal Kenya towns, has a mix of both Christians and Muslims, with some residents practicing indigenous religions. The area is characterized by high rates of sexual and gender-based violence (SGBV) and early/teenage pregnancy [23, 24].

Kisumu, located in western Kenya, is the fourth county. This peri-urban county is characterized by a blend of urban and rural areas, and it is also known for its high prevalence of HIV [25, 26]. The population is ethnically diverse, with several communities living in the area. Some of the prominent ethnic communities include the Luo and Kisii. The Luo community is particularly significant due to its historical and cultural ties to the region.

### Analytic sample

The study leverages two rounds of longitudinal quantitative data and two rounds of qualitative data collected in 2020 and 2022. The quantitative baseline data was collected between June and August 2020. A follow-up round targeting all adolescents interviewed in 2020 was collected between February and March 2022. The qualitative interviews were conducted in November 2020 and April 2022. A detailed description of the sample selection has been published elsewhere [27]. The analytical sample includes all respondents aged 10–19 who were in primary school (grades 1 to 8) or lower secondary school (forms 1 and 2, equivalent to grades 9 and 10) before the COVID-19 school closures in 2020. The same respondents were re-interviewed two years later in 2022, a time by which they should have progressed two grades–e.g., those in form 2 (grade 10) should have progressed to form 4 (grade 12), and those in grade 1 should have advanced to grade 3. Only respondents successfully re-interviewed in 2022 were included in the analysis (See Table 1). Respondents for the qualitative interviews were drawn from the quantitative sample with additional stakeholders included as described further below.

**Table 1:** Analytic sample.

	Female participants	Male participants	Total
Total aged 10–19 interviewed in 2020	2814	1106	3920
Included in the analytical sample	1255	543	1798
Eligible for analytical sample but not interviewed in 2022	469	245	714
Excluded from the analytical sample			
Attending grade 11 or higher pre-Covid	645	161	806
Out of school pre-Covid	439	149	588
Unknown school status pre-Covid	6	8	14

Note: The "Eligible for analytical sample but not interviewed in 2022" category includes participants who were lost to follow-up during the study period.

### Quantitative approach

We analyzed data on factors associated with school retention post-COVID-19 school closures. The dependent variable was school retention coded using a binary response that is equal to 1 if the adolescent was attending school in 2022, among adolescents who were in school before the pandemic.

Covariates measured in 2020 included the following: (i) respondent’s age at last birthday, defined as a continuous variable; (ii) household loss of income due to COVID-19 defined as a binary variable that is equal to 1 if the household lost income and 0 otherwise, (iii) time spent on household tasks relative to pre-Covid, defined as a binary variable that is equal to 1 if the respondent spent more time on household tasks since COVID-19, and 0 if the time spent was about the same or less; and (iv) food security (skipping meals), defined as 1 if a respondent skipped a meal either daily, once a week, or a couple of times in a week and 0 if otherwise. The following covariates measured in 2022 were also examined: (i) involvement in income-generating activities, defined as 1 if the respondent was engaged in an income-generating activity before the reopening of schools in 2022 and 0 if otherwise (ii) access to the internet, defined as a binary variable that is equal to 1 if the respondent had used the internet at least once in the past month, whether at home, school, or elsewhere, and 0 if otherwise; (iii) access to a smartphone, defined as a binary variable that is equal to 1 if the respondent owned or had regular access to a smartphone with internet capability, and 0 if otherwise; (iv) pregnancy (pregnant or recently had a baby), defined as a binary variable that is equal to 1 if a female respondent was currently pregnant at the time of the survey in 2022 or had given birth in the 12 months preceding the survey (i.e., between 2021 and early 2022) and 0 if otherwise; (v) marriage status defined as a categorical variable that is equal to 0 if a female respondent is not married, 1 if married before COVID-19 and 2 if married after COVID-19. In addition, grade attainment pre-Covid, measured as a continuous variable, and study site/county were included as confounding factors in multivariate models.

Descriptive statistics were used to explore how key variables were distributed by gender. To examine the factors associated with school retention we first assessed for multicollinearity by checking the correlations among the variables of interest. Two factors, access to a phone and access to a smartphone, were dropped as they were highly correlated with access to the internet. We thus retained access to the internet variable which had the highest correlation with the outcome variable as compared to owning a phone and access to a smartphone. Pregnancy and marriage status factors were also not included in the regression models because of the small numbers of those who responded yes to being pregnant or having a baby and of those who got married after the pandemic. A bivariate association between predictor variables and school retention in separate binary logistic regression models by gender was then examined. This was followed by fitting full logistic regression models to control for the confounders and identify independent factors associated with school retention from variables that demonstrated significant bivariate associations with school retention. Adjusted odds ratios (aOR) and 95% confidence interval (CI) for each covariate were reported.

### Qualitative approach

Qualitative data was collected to complement the quantitative work and to deepen understanding of the issues of interest. Following the quantitative survey, face-to-face in-depth interviews were conducted with a diverse group of participants across the four counties. These included 64 adolescents from the existing cohorts, 25 parents of participating adolescents, and 21 key informants (village guides, teachers, and local administrators). Interviews were conducted in the relevant local language and covered various topics that were pertinent to the research objectives including but not limited to school retention, pregnancy, child marriage, domestic responsibilities, school environment, learning quality, and food security. Interviews were conducted by interviewers trained in qualitative data collection, research ethics, and safeguarding protocols for working with adolescents. To ensure data accuracy, routine quality checks were performed throughout the process. All data was de-identified prior to analysis to maintain participant confidentiality.

All interviews were audio-recorded, transcribed with simultaneous translation, and independently verified for quality and accuracy by validators who were proficient in the local language. The validation process entailed validators listening to the audio recordings while reading the translated transcripts and, where necessary making edits in the Word document using tracked changes; they would then discuss with the original transcriber to address any discrepancies and agree on the most accurate translation that conveyed the intended meaning of the respondent. Only after this process were the transcripts analysed. Data was managed using NVivo software and analysed using a thematic analysis approach. This entailed immersion in the raw data through extensive familiarization with the interview transcripts and field notes. A coding scheme was then consultatively developed to categorize emerging themes. The coding process involved splitting and rearranging the data from all the respondents according to thematic content. The findings from this process were then organized into county-specific charts to enable comparison. Data analysis was conducted by a team of trained researchers ensuring consistency through regular discussions and using Krippendorff’s alpha to assess coding reliability. Discrepancies were resolved in consensus meetings, where the team reviewed and aligned their coding decisions.

The ethical approval for this study was obtained from the Population Council Institutional Review Board (p936) and AMREF Ethical Scientific Review Committee (P803/2020). Additionally, the study acquired a research permit from the National Commission for Science, Technology, and Innovation (P/22/16531). Participants over the age of 18 consented after being given full information, while those under 18 assented to participate and their parent or legal guardian’s consent was sought. Interviews were conducted in person while strictly adhering to COVID-19 protocols, including maintaining social distancing, wearing masks, and ensuring proper sanitation practices.

## Results

In this section, the findings have been presented by each of the factors associated with school retention, starting with quantitative findings and complementing with insights from the qualitative data. In addition, we present qualitative findings on the enablers of school retention.

### Factors associated with school retention by gender

Table 2 shows the sociodemographic characteristics of respondents and factors associated with school retention by gender. The participant’s baseline age (before school closures in 2020) ranged from 10–19 with an average age of 14.2 and 16.1 for males and females respectively. At the start of the pandemic, 57% of the male and 61% of the female participants were from households that had either partially or completely lost income. Fifty-six percent of the male adolescents and 65% of the female adolescents reported having spent more time on household tasks during the pandemic (and school closures) compared to before. Thirty-five percent and 44% of the male and female adolescents respectively reported having skipped meals at the start of the COVID-19 pandemic. Fifteen percent of male and 9% of female adolescents reported having engaged in income-generating activities in the past month; 23% of male and 18% reported having accessed the internet whereas 10% of each gender reported having access to a smartphone. For female adolescents, 3% reported either being pregnant or recently having a baby, 1.3% reported having married after COVID-19 whereas 0.3% reported having married before COVID-19.

**Table 2:** Characteristics of the respondents and factors associated with school retention by study gender.

	Male	Female
Analytic sample	n = 543	n = 1255
***Covariates measured in 2020***		
Age		
Mean	14.2	16.1
SD	2.1	1.8
Household loss of income		
No	43%	39%
Yes	57%	61%
Time use on household tasks		
More	56%	65%
Less or about the same	44%	35%
Skipping meals		
No	65%	56%
Yes	35%	44%
***Covariates measured in 2022***		
Earning income in the past one month		
No	85%	91%
Yes	15%	9%
Access to internet		
No	77%	82%
Yes	23%	18%
Access to smartphone		
No	90%	90%
Yes	10%	10%
Pregnant or recently had a baby		
No		97%
Yes		3%
Marriage status		
Not married		98.4%
Married after covid		1.3%
Married before covid		0.3%

Note: Percentages represent the proportion of respondents within each gender group. "Pregnant or recently had a baby" and "Marriage status" were assessed for females only.

As noted in Table 1, a considerable number of baseline respondents who were in school pre-Covid and are within the relevant age range were not included in this analysis as they lost to follow-up in 2022. To assess potential bias due to attrition, we compared baseline characteristics of respondents in our analytical sample and respondents who were lost to follow-up in 2022 (see Table 3). The results indicate that respondents lost to follow-up differ significantly in certain characteristics. For female respondents, there is a significant difference in age between those retained and lost to follow-up (p = 0.0111), with those lost to follow-up being slightly older on average. Study site distribution also shows significant differences for Nairobi (p < .001), Kilifi (p = 0.019), Kisumu (p = 0.001), and Wajir (p < .001), suggesting variations in attrition rates by location. For male respondents, there were no significant differences in age (p = 0.3106) or grade level (p = 0.3948). However, there were significant differences in study site distributions for Nairobi and Wajir (both p < .001), indicating possible location-related attrition patterns. To mitigate potential bias due to these differences, the study site was included as a confounding variable in the multivariate models.

**Table 3:** Comparison of baseline characteristics between the analytical sample (group = 1) and respondents lost to follow-up (group = 0) in 2022.

	**Female**			**Male**		
**Variable**	**Group (N)**	**Mean (SD)**	**P**	**Group (N)**	**Mean (SD)**	**P**
Age	0(469)	15.8(1.99)	0.0111	0(245)	14.4(2.17)	0.3106
	1(1255)	16.13 (1.83)		1 (543)	14.24(2.12)	
Grade Level	0 (469)	8.54(1.66)	0.5653	0 (245)	6.98(2.11)	0.3948
	1 (1255)	8.59(1.69)		1 (543)	6.83(2.31)	
**Study site**	**Group**	**Mean**	***P***	**Group (N)**	**Mean**	***P***
Nairobi	0 (469)	0.32	< .001	0 (245)	0.28	< .001
	1 (1255)	0.23		1 (543)	0.11	
Kilifi	0 (469)	0.33	0.019	0 (245)	0.32	0.469
	1 (1255)	0.39		1 (543)	0.29	
Kisumu	0 (469)	0.14	0.001	0 (245)	0.16	0.777
	1 (1255)	0.08		1 (543)	0.15	
Wajir	0 (469)	0.21	< .001	0 (245)	0.25	< .001
	1 (1255)	0.30		1 (543)	0.45	

Note: p-values were calculated using t-tests for continuous variables (age and grade) and proportion tests for the binary variable (each study site was treated as a binary variable)

Table 4 shows the results of separate binomial logistic regression models of school retention by gender. As reflected in the models, the following factors were significantly associated with school retention post-COVID-19 school closures for female adolescents: age (odds ratio (OR) = 0.72, 95% confidence interval (CI): 0.64, 0.82), household loss of income (OR = 1.67, 95% CI: 1.14, 2.42), earning income (OR = 0.27, 95% CI:0.17, 0.43), and internet access (OR = 0.56, 95% CI:0.37, 0.87). Time use and skipping meals did not exhibit a significant association with school retention.

**Table 4:** Bivariate logistic regressions associated with school retention.

	FEMALES				MALES			
Retention	OR	*P*	95% CI		OR	*p*	95% CI	
**Age**	0.72	<0.001	0.64	0.82	0.85	0.010	0.75	0.96
**Loss of income**								
No	1				1			
Yes	1.67	0.008	1.14	2.42	0.88	0.626	0.52	1.50
**Time use**								
More	1				1			
Less or about the same	0.28	0.239	0.85	1.91	1.68	0.067	0.96	2.92
**Skip meals**								
No	1							
Yes	0.91	0.632	0.63	1.33	0.86	0.583	0.50	1.48
**Earning income**								
No	1				1			
Yes	0.27	<0.001	0.17	0.43	0.27	<0.001	0.15	0.50
**Access to internet**								
No	1				1			
Yes	0.56	0.008	1.14	2.42	0.88	0.626	0.51	1.50

Note: Odds ratios (OR) represent the odds of school retention associated with each covariate, with reference categories specified; p-values <0.05 indicate statistically significant associations, and 95% confidence intervals (CI) provide the range of plausible values for the odds ratios, with analyses conducted separately for females and males.

For male adolescents, age (OR = 0.85, 95% CI: 0.75, 0.96) and earning income (OR = 0.27, 95% CI:0.15, 0.50) were significantly associated with school retention post-COVID-19 school closures. Household loss of income, time use, skipping meals, and access to the internet did not have a significant association with school retention for male adolescents.

Table 5 shows the results of multivariate logistic regression models for school retention for both female and male adolescents. Only factors that were identified by bivariate logistic regressions as having a significant association of p< = 0.05 with the outcome variable of school retention after COVID-19 school closures were included in the models. The study site (county) and pre-Covid grade levels were included in the model as control variables.

**Table 5:** Multivariate logistic regression models for school retention adjusted for study site and baseline grade.

	FEMALES				MALES			
Retention	aOR	*P*	95% CI		aOR	*p*	95% CI	
**Age**	0.76	<0.001	0.67	0.87	0.88	0.127	0.76	1.03
**Loss of income**								
No	1				1			
Yes	1.40	0.147	0.89	2.23	0.40	0.006	0.21	0.76
**Earning income**								
No	1				1			
Yes	0.27	<0.001	0.16	0.46	0.07	<0.001	0.02	0.19
**Access to internet**								
No	1				1			
Yes	0.55	0.010	0.35	0.87	0.96	0.923	0.47	1.58

Note: Adjusted odds ratios (aOR) represent the likelihood of school retention associated with each covariate, adjusted for study site and baseline grade, with reference categories specified. P-values <0.05 indicate statistically significant associations, and 95% confidence intervals (CI) reflect the precision of the estimates, analyzed separately for females and males

### Age

Age was significantly associated with school retention for female adolescents. An increase in age was associated with a 24% decrease in the odds of a female adolescent being retained in school (adjusted odds ratio (aOR) = 0.76, 95% CI: 0.67, 0.87). There was however no significant association between age and school retention for male adolescents (aOR = 0.88, 95% CI: 0.76, 1.03). The results suggest that older female adolescents were more likely to drop out of school as compared to young female adolescents. The qualitative insights indicate that pregnancy and motherhood make it difficult for older adolescent girls to complete secondary school as illustrated in the following quote;

*“I feel very bad when I see others going to school and I am at home taking care of the child that pains me a lot because those who are going to school will have a better life….”*

***Adolescent girl*, *15–19 years*, *Nairobi***

### Household loss of income

The findings on household loss of income highlight a significant link between household income loss and school retention for male adolescents, with a 60% decrease in retention odds (aOR = 0.40, 95% CI: 0.21, 0.76) for those from households that experienced a loss of income at the start of the pandemic. Qualitative insights reveal that this financial strain had a profound impact on male adolescents’ mental health, contributing to increased anxiety, stress, and worry about their ability to complete their studies. Many male adolescents expressed concern about their parent’s inability to pay school fees, often leading them to drop out to support their families financially. This decision was especially common after schools reopened, as financial pressures were compounded by parents’ diminished income due to the pandemic. Beyond the impact on school fees, male adolescents also faced mental strain over the family’s general financial hardship, which led some to prioritize immediate family needs over their education. In some cases, young men shared that they were compelled to seek work to contribute to household income, thus foregoing school altogether. One adolescent described this sentiment, stating,

*“Our parents are suffering right now. So, you know, you find that your parent has returned home [at the end of the day] without money. You sympathize with how they struggle for you to be able to eat. So, this will keep on being on your mind to the extent that you get stressed.”*

Adolescent boy 15–19 years, Kisumu

This quote illustrates the internal conflict and stress these adolescents experienced, feeling both empathy for their parents and the weight of their educational disruption.

### Earning income

Engaging in income-generating activities was significantly associated with school retention for both female and male adolescents. Those adolescents who earned income were less likely to retain their schooling post-COVID-19 school closures. Engaging in income-generating activities was associated with a 73% decrease in the odds of a female student school retention (aOR = 0.27, 95% CI: 0.16, 0.46) and a 93% decrease in the odds of a male student school retention (aOR = 0.07, 95% CI: 0.02, 0.19). This implies that adolescents who needed to generate income were at higher risk of dropping out of school. From the qualitative findings, we also observed that engagement in income-generating activities was a key barrier, especially for the adolescent boys who engaged in these activities during the school closures. For these boys, earning an income (and the allure of it) was deemed far more valuable than going back to school. Indeed, resuming school was perceived as a hindrance to earning money, especially where there had been a loss of parental income because of the pandemic and related restrictions.

*“The boy lost interest in learning (school). He stayed home for so long until other boys started telling him they should look for things to sell so that they get some little money to maintain themselves. Because at that time I would go search for casual jobs and would not get any. Then there were no jobs, we were not even allowed to work in people’s homes.”*

Parent, Nairobi

The need to earn income was also sometimes linked to adolescent pregnancy. That is, an adolescent boy who accepted responsibility for a girl’s pregnancy, would then be forced to drop out of school to provide for his young family as illustrated in the quote below;

*“Even if you take him to school and yet he has two kids and they want food…the boy is forced to [drop out of school] and venture into things like bodaboda (motorbike public service transport for income generation) because he has a wife and kids to take care of.”*

Parent, Kilifi

### Internet access

Among female adolescents, internet access was negatively associated with school retention post-COVID-19 school closures. Female adolescents with internet access were less likely to be retained in school (aOR = 0.55, 95% CI: 0.35 0.87). The results show that internet access was associated with a 45% decrease in the odds of an adolescent girl being retained in school. The results suggest a gender-specific association between internet access and school retention after COVID-19 school closures. This gender-specific association between internet access and school retention is further illustrated by a respondent who shared the following:

*“Sometimes when you see people going to school, you feel like you have been isolated. And also, sometimes there is nothing that you are doing, you will watch a movie when you are done you sleep. Because there is no one you can visit since everyone is in school even if you chat with them, someone tells you is in school. So that time you feel like you are the odd one out.”*

Adolescent girl, 15–19 years, Nairobi

### Pregnancy and child marriage

We did not include pregnancy and child marriage in the multivariate analysis due to low numbers. However, pregnancy, child marriage, childbirth, and associated childcare responsibilities were identified from the qualitative data as significant factors leading to school dropout, especially among older female adolescents aged 15 to 19 years. This included discomfort due to pregnancy as illustrated in the quote below.

*“For now, I still have some bit of stress. I became pregnant so learning is sometimes very challenging… I cannot sit down for long and then at night, I have to sleep early. Meaning, sitting for long at night while studying has become uncomfortable.”*

Adolescent girl 15–18 years, Kisumu

Relatedly, shame and stigma (from peers, teachers, and the broader community) associated with teenage pregnancy, were a deterrent to girls returning to school. Even where parents expressed support for their daughters to continue schooling after giving birth, sometimes the adolescent mothers had lost interest and enthusiasm about resuming their schooling; exacerbated by the humiliation they felt for having fallen pregnant. A stakeholder and an adolescent mother described how both ‘self-shame’ and ridicule from others such as teachers, kept girls away from school post-COVID-19 school closures, stating;

*“There are those girls that got pregnant, because of staying idle with nothing to do. So, some felt ashamed to go back to school and decided that the only option was to stay at home.”*

Stakeholder, Nairobi.

“I don’t get along with some teachers, like one male teacher. When he gets to class, he just talks ill of me. When he is explaining something in class, he tells other students that there are others who know about it like me because I already gave birth, and I can tell more about it. I do feel bad, but I don’t say anything. My parents also, especially my father always discourages me. He says I was cheated [by the child’s father]and now I can’t grasp anything while studying.”

Adolescent mother 15–19 years, Kilifi.

Specifically, in Wajir County, early marriage was noted as a key factor in failure to return to school after the pandemic-related closures. As illustrated below, it was reported that during the extended school closures, some parents in Wajir married off their adolescent girls, as it was assumed that schools would never reopen. Consequently, these girls never returned to school.

*“Some schoolgirls were forced to marry. Parents thought that schools would never reopen after the coronavirus, so they married off their daughters.”*

Adolescent girl 15–18 years, Wajir

### Additional findings from the qualitative data

It was also observed in Wajir County–where ownership of livestock is highly valued and associated with wealth–that some parents undervalued the importance of education in comparison to livestock rearing. As a result, they chose to have their daughters stay at home (even after schools reopened) so that they could take care of livestock and attend to other domestic chores.

*They thought that schools would never reopen and left to live with their animals far away from the village. Since there was no learning, they took their children with them to look after their animals for them.”*

Adolescent girl 10–14 years, Wajir

Drug and alcohol use was another key emergent theme related to the failure of boys to re-enrol and/or remain in school. This was especially prevalent in the urban and peri-urban counties (Nairobi, Kisumu, and Kilifi). As illustrated below, many respondents (including the adolescents themselves) raised this as a major concern, adversely impacting male children in their communities.

*“Due to the holiday [school closures], many of them [boys] joined these groups that deal in marijuana and [did not return to school]. They [now] see themselves as untouchable. Many of them ran away from home…they have not returned to their homes.”*

Adolescent boy, 15–18 years, Nairobi

Across all four counties, there were cross-cutting issues for both boys and girls that hindered school re-entry post-pandemic school closures and/or retention in school. One such factor was the lack of school fees, which emerged strongly as a reason for school dropout. This was exacerbated by parental job losses as a result of the pandemic and related restrictions.

*“One challenge is that after COVID-19, some adolescents have not gone back to school. While those who completed their studies are jobless and others have engaged in drugs and substance abuse due to these challenges. Also, lack of school fees is a challenge. There are some Adolescents who have performed well in their exams but may not join secondary school or drop out because of school fees.”*

Parent, Wajir

Despite many constraints, there were, however, enablers to adolescent school re-enrolment and retention. One key enabler was the local administration. As illustrated below, some respondents noted that the local administration, led by chiefs and village elders, played an active and crucial role in ensuring that most adolescents returned to school when schools reopened.

*“Many stayed at home but when they heard that the chief was going around checking, that is when some parents took their children back to school. There are some who did not take their children back, but there was a time when the chief went around looking for children who were not going to school, and the parents were forced to take them to school.”*

Parent, Kilifi.

The village guides were, however, not always successful as some parents withheld or gave false information on the whereabouts of their children. This was especially the case where adolescent children were involved in income-generating activities that supported the family.

*“As village guides we mostly get this challenge because you will follow a parent and ask them why their child has not yet gone back to school…they will tell you that their child is in a school that is far from here, and when you do an investigation you will find out that this child is in fact at work and not at school while the parent lied to you. So, returning this child back to school becomes difficult.”*

Stakeholder, Kilifi

## Discussion

This mixed methods study explored factors associated with school retention among marginalized adolescents following the COVID-19 school closures in Kenya. The study identified various factors that hindered (or enabled) school retention, with gendered nuances also playing a role. Findings from the quantitative analysis showed that earning income was significantly associated with low school retention for both female and male adolescents. However, age and internet access were specifically significant for female adolescents.

The correlation between age and school retention aligns with common observations that older students often contend with heightened responsibilities and pressures, potentially impeding their ability to sustain their education. This age-related pattern is consistently reflected in various studies, underscoring the heightened vulnerability of older adolescents concerning school retention during pandemics. According to the Kenya Demographic Health Survey of 2022, school dropout rates are notably elevated among older adolescents, particularly females, compared to their younger counterparts [28]. Kumar and others’ recent research on determinants of school dropouts among adolescents in India further supports these findings, revealing a peak in school dropout rates among older adolescents [10]. Notably, studies indicate that these dropouts disproportionately affect the most vulnerable learners.

The findings on household income loss and male adolescent school retention underscore how financial strain during the pandemic impacts young men’s education. The observed 60% decrease in retention odds for male students from households with household loss of income suggests that, in many cases, boys were expected to support family income needs, leading them to prioritize work over school. This trend reflects similar findings from a study by Di Maio and Nisticò in 2019 on the effect of parental loss on children’s school dropout in the Occupied Palestinian Territories [29]. Their findings indicate that children are 9 percentage points more likely to drop out of school if their parents experience job loss. As observed by Kumar and colleagues, younger boys who participated in paid work were 6.67 times more likely to drop out of school compared to their peers who did not engage in such work [30]. Economic crises often force young boys to become "reserve income earners" in their families, especially in low-income contexts. Miguel and Mobarak also noted that financial disruptions caused by the pandemic disproportionately impacted male adolescents’ educational trajectories, with increased dropout rates where income contributions were needed at home [31].

The findings on the negative association between engaging in income-generating activities and school retention concur with previous studies undertaken during the pandemic. For instance, a report by the International Labour Organization (ILO) in 2020 revealed that the COVID-19 crisis pushed millions of children into child labor, reversing years of progress [32]. Even though previous studies also reveal a negative association between earning income and school retention for adolescents [33], the situation seems to have been worsened by the COVID-19 pandemic. Lockdown measures and economic hardships during the pandemic forced many families to rely on additional sources of income, often at the expense of their children’s education.

Specifically for boys, drug and alcohol abuse emerged from the qualitative data as a key factor to school dropout in addition to engagement in income-generating activities. Studies from many other contexts on the relationship between school dropout and drug and alcohol abuse agree with our findings; that is, alcohol and drug use lead to school dropout. For example, in his 2018 review of different studies on the relationship between school dropout and substance, Valkov found that school dropout predicts substance use [34]. Furthermore, other studies show that school dropout precedes drug and alcohol use. This aligns with our study findings which imply that the extended COVID-19 school closures (in some ways a ‘forced drop out’ from school), precipitated the use of drugs and alcohol especially by male adolescents. Tice and Van Horn for example in their 2017 study undertaken in the United States of America found that students who drop out of school are more likely to engage in drug and alcohol use [35].

Our study unveils surprising results that internet access during the COVID-19 pandemic did not translate to school retention among marginalized female adolescents in Kenya. Although we did not find a similar study supporting our findings, our study seems to suggest that adolescent girls are more likely to face negative unintended consequences while accessing the internet as compared to boys. The qualitative quote offers valuable context to these findings. The adolescent respondent describes feelings of *isolation* and *disconnection* from peers who continued attending school, despite being able to engage online. Rather than using the internet for educational purposes, the respondent mentions turning to entertainment, such as watching movies, which ultimately deepened feelings of exclusion and lack of purpose. This emotional withdrawal may have led to further disengagement from school, reinforcing the negative relationship between internet access and school retention seen in the quantitative data. Similar observations were made by Okadia in 2021, who noted that adolescents who accessed the internet in Kenya during COVID-19 school closures were negatively affected by the content which they interacted with due to a lack of proper guidance from their parents [36]. This gender-specific discrepancy in digital literacy, coupled with the potential presence of distractors or negative online influences may have significantly hampered the quality of engagement that adolescent girls experienced during the pandemic. Our findings emphasize the need for targeted interventions addressing gender-specific barriers to effective online learning.

The relationship between adolescent pregnancy and school dropout during the COVID-19 pandemic has been the subject of extensive research. Our findings are consistent with previous studies that have demonstrated the detrimental impact of pregnancy on educational outcomes during this unprecedented time. For instance, a study by Zulaika and others in 2022 on the effect of COVID-19 lock down on adolescent pregnancy and schooling in Kenya shows that the pandemic exacerbated school dropout among adolescent girls [16].

Early marriage often disrupts girls’ education and can have long-lasting negative effects on their prospects. Our study has shown that adolescent girls who were pregnant or had recently given birth during COVID-19 school closures were less likely to be retained in school. These findings are similar to those from previous studies. For instance, a study by Kidman and others in 2022 on school resumption following COVID-19 closures in Malawi alludes that girls who were married or had children during the COVID-19 lockdown were less likely to return to school after the lockdown [37]. A study by Dessy and others in 2021 on COVID-19 and children’s school resilience in Nigeria also shows that the pandemic exacerbated child marriage and the teen mothers were therefore more likely to discontinue their schooling [38].

One key enabling factor for school retention that was highlighted by the qualitative findings was the important role played by local administration, such as chiefs and village elders. This finding is consistent with observations from other African contexts, where local leadership and key stakeholders have been shown to positively promote education. For instance, research conducted by Mwelwa and others in 2020 in Zambia highlighted the importance of involving traditional leaders in educational initiatives to stop child marriage and increase school attendance and retention among adolescent girls [39]. In this Zambian study, the measures implemented included awareness campaigns, partnering with support groups, and adjusting the timing for traditional ceremonies to avoid disrupting school attendance.

## Conclusion

The findings of this study illuminate factors that hinder school retention among marginalized adolescents in the context of a crisis such as the COVID-19 pandemic. The results point to a need for targeted policy and programme interventions to ensure that the most vulnerable attain educational goals and do not ‘fall through the cracks’ of the education system following a crisis.

The age-related decrease in school retention among female adolescents highlights the need for tailored interventions that recognize the unique challenges faced by older adolescent girls including increased responsibilities and life transitions. To support the educational aspirations of pregnant and parenting adolescents, comprehensive and sensitive support mechanisms should be put in place, including access to healthcare, childcare services, and educational opportunities tailored to their needs. For instance, the Ministry of Education’s 4Ts initiative in Kenya—Track, Trace, Talk, and Return—successfully engages families to inform them of school re-entry policies, ensuring that young mothers are supported in returning to school [40]. Similarly, the Camfed Tanzania Initiative provides targeted support to marginalized girls, including mentorship and bursary schemes, which have been instrumental in keeping older adolescent girls in school despite the challenges posed by early marriages and caregiving responsibilities [41].

Based on our findings on internet access, we recommend supporting female adolescents in marginalized communities by offering practical skills on focused online learning platforms and guiding them on accessing appropriate educational materials. This approach aligns with UNESCO’s recommendations for closing the digital gender gap by establishing community learning hubs equipped with internet access and digital training for girls [8]. By establishing these localized learning hubs, female adolescents can be significantly impacted, ensuring equitable access to educational resources and improving their overall learning experiences.

To mitigate the risk of dropping out of school due to lack of school fees, interventions should be designed to reduce the adolescents’ financial burdens so that they can concentrate on their schooling. Financial barriers often disproportionately affect marginalized adolescents, particularly girls, making it essential to implement targeted support systems. For instance, cash transfer conditioned on school attendance is one of the approaches that has been shown to work in ensuring that marginalized adolescents are kept in school, promoting not only retention but also enhancing academic performance and reducing dropout rates [42]. These financial incentives alleviate the immediate pressures families face, allowing students to focus on their education without the distraction of economic hardships. This approach has been associated with reducing and/or preventing early marriages, as families are less likely to view their daughters as economic assets when they can contribute to educational expenses [43, 44]. Furthermore, integrating these cash transfer programs with complementary services such as mentorship and educational resources can enhance their effectiveness, creating a supportive environment that encourages adolescent to pursue their goals [45].

There is a need to address the social and emotional well-being of adolescents during times of crisis and provide them with support to prevent drug and alcohol use and the associated negative consequences. An example from Kenya’s *Wasichana Wetu Wafaulu* program illustrates the value of community-based psychosocial support for adolescents, which has successfully reduced risky behaviors and improved educational outcomes by strengthening the role of community champions in promoting safe spaces and mentorship programs [46]. Strengthening community engagement between local educational authorities and the local administration, chiefs, and village elders will ensure a unified effort to promote education retention among marginalized adolescents. For instance, the *Room to Read* program in Tanzania, which actively involves community leaders and parents in supporting girls’ education, has demonstrated how community-level engagement can enhance school attendance and retention, particularly for vulnerable girls facing crises [47]. By adopting a holistic approach to supporting adolescents’ educational aspirations, policymakers and educational stakeholders can contribute to a more inclusive and resilient educational system for all adolescents, even in the face of unprecedented challenges like the COVID-19 pandemic.

## Limitations

The findings of this study provide valuable insight into the factors influencing school retention among marginalized adolescents in Kenya following the COVID-19 pandemic. However, it is important to acknowledge some limitations that should be considered when interpreting and applying these findings. A key limitation is that the majority of girls in this study had participated in girls’ empowerment programs so there is a good chance that the most marginalized adolescents might have been missed out. Second, the sample of boys is limited to boys residing in households with adolescent girls, thus a younger sample relative to the girls’ sample. Third, the focus on marginalized adolescents in Kenya may limit the generalizability of the findings to other regions or populations with differing socio-economic, cultural, and educational contexts.

Fourth, not all relevant variables influencing school retention were considered in the quantitative analysis, hence missing important contributors. We however explored other potential factors through qualitative data. Fifth, the quantitative data were self-reported and collected through phone surveys which could lead to response bias and also missing out on the most marginalised adolescents with no phone access. We however tried as much as possible to capture rich contextual insights from qualitative data which might have been missed out from quantitative data. Additionally, differential attrition by age, particularly among female respondents, may have introduced bias that limits the generalizability of the findings.
